# A Novel Prognostic Model for Acute Myeloid Leukemia Based on Gene Set Variation Analysis

**DOI:** 10.1155/2022/7727424

**Published:** 2022-11-21

**Authors:** Shuai Zhang, Qianqian Wang, Haoran Xia, Hui Liu

**Affiliations:** ^1^Department of Hematology, Beijing Hospital, National Center of Gerontology, Institute of Geriatric Medicine, Chinese Academy of Medical Sciences, Bejing, China; ^2^Graduate School of Peking Union Medical College, Chinese Academy of Medical Sciences, Beijing, China; ^3^Peking University China-Japan Friendship School of Clinical Medicine, Bejing, China; ^4^Department of Urology, Beijing Hospital, National Center of Gerontology, Institute of Geriatric Medicine, Chinese Academy of Medical Sciences, Bejing, China

## Abstract

Acute myeloid leukemia (AML) is a malignant hematological malignancy with a poor prognosis. Risk stratification of patients with AML is mainly based on the characteristics of cytogenetics and molecular genetics; however, patients with favorable genetics may have a poor prognosis. Here, we focused on the activity changes of immunologic and hallmark gene sets in the AML population. Based on the enrichment score of gene sets by gene set variation analysis (GSVA), we identified three AML subtypes by the nonnegative matrix factorization (NMF) algorithm in the TCGA cohort. AML patients in subgroup 1 had worse overall survival (OS) than subgroups 2 and 3 (*P* < 0.001). The median overall survival (mOS) of subgroups 1–3 was 0.4, 2.2, and 1.7 years, respectively. Clinical characteristics, including age and FAB classification, were significantly different among each subgroup. Using the least absolute shrinkage and selection operator (LASSO) regression method, we discovered three prognostic gene sets and established the final prognostic model based on them. Patients in the high-risk group had significantly shorter OS than those in the low-risk group in the TCGA cohort (*P* < 0.001) with mOS of 2.2 and 0.7 years in the low- and high-risk groups, respectively. The results were further validated in the GSE146173 and GSE12417 cohorts. We further identified the key genes of prognostic gene sets using a protein-protein interaction network. In conclusion, the study established and validated a novel prognostic model for risk stratification in AML, which provides a new perspective for accurate prognosis assessment.

## 1. Introduction

Acute myeloid leukemia is a heterogeneous myeloid neoplasm, characterized by uncontrolled proliferation and impaired differentiation of myeloid blasts. Despite the development of novel antileukemic drugs, the prognosis of AML has remained poor, with a 5-year overall survival rate of less than 30% [1]. Thus, it is of great importance to identify prognostic biomarkers to predict prognosis and better understand the pathogenesis of AML. The European LeukemiaNet (ELN) recommended that patients should be stratified according to their cytogenetics and molecular genetics, which are the main basis of risk stratification for AML, and treated with chemotherapy or allogeneic stem cell transplantation, respectively [2]. However, patients with favorable genetics may have a poor prognosis [3], which necessitates new methods to improve the prognosis assessment system.

Previous studies on prognosis assessment in AML have mainly focused on a single gene or a group of genes related to specific biological processes [4–6], instead of comprehensively integrating multiple pathways in AML expression gene sets. For example, autophagy-related signatures [7], ferroptosis-related genes [8], and immune-related signatures [9] were reported to be related to the prognosis of AML, respectively. Here, we applied gene set variation analysis (GSVA) to detect subtle changes in the pathway within an entire gene expression set [10], and then the patients were classified according to the enrichment score (ES) of gene sets in an unsupervised manner.

To our knowledge, subtypes based on the activity changes of gene sets in AML have not been determined. In the present study, we identified three subtypes of AML based on the activity changes of hallmark and immunologic gene sets in AML and established a prognostic model using TCGA gene expression data. Subsequently, the prognostic model was further validated in GEO cohorts. Our study provides a novel perspective for the prognostic model of AML by identifying the activity changes of gene sets in the AML population instead of focusing on changes in a single gene.

## 2. Materials and Methods

### 2.1. Data Collection and Processing

One hundred and twenty-five patients were used as the training cohort (patients with AML M3 were excluded), and their fragments per kilobase per million mapped reads (FPKM)-normalized RNA-Seq data and clinical information were downloaded from the TCGA database via the GDC data portal (https://portal.gdc.cancer.gov/repository). Two gene expression profiles of AML, GSE146173 (*n* = 246) and GSE12417 (*n* = 242), and the corresponding probe annotation platform files (GPL18460 and GPL96) were downloaded from the GEO (https://www.ncbi.nlm.nih.gov/geo/). Data normalization and log2 transformations were performed on all gene expression datasets.

### 2.2. Gene Set Variation Analysis

A total of 4922 ImmuneSigDB gene subsets of C7 and hallmark gene sets were downloaded from MSigDB (https://www.gsea-msigdb.org/gsea/msigdb/index.jsp) for single sample gene set variation analysis (ssGSVA) with the GSVA R package. We used GSVA to evaluate the changes in pathway activity over the TCGA cohort and ES to reveal the activation degree [10]. Accordingly, the gene expression matrix from TCGA and GEO cohorts was transformed into the pathway ES matrix, which was uploaded as Supplementary Tables 1 and 2.

### 2.3. Nonnegative Matrix Factorization (NMF) Algorithm and Supervised Hierarchical Clustering

In the training cohort, Cox regression analysis was used to filter the gene sets related to prognosis (R package CancerSubtypes) with *P* < 0.001. Subsequently, patients were divided into three types (C1-3) by NMF based on the ESs. According to the clustering results of the training set (TCGA), the 100 most significantly upregulated gene sets in each cluster (if less than 100, all gene sets were selected) were selected as subgroup features. Accordingly, patients from the GSE146173 dataset were used as an independent validation cohort for supervised hierarchical clustering. The relevant files generated during the calculation were uploaded as Supplementary Tables 3–5.

### 2.4. Prognostic Model Construction and Validation

Differential gene sets of each two cluster were analyzed with R package limma, filtered by *P* < 0.05 and |logFC| > 0.02, and intersected subsequently. A univariate Cox regression analysis was used to screen gene sets associated with survival, filtered by *P* < 0.05 (R package survival). The least absolute shrinkage and selection operator (LASSO) regression method was performed to further screen prognostic gene sets and obtain their corresponding coefficients (R package glmnet). The “maxit” was set to 1000. The final risk score was calculated as follows: risk score = ∑_*i*_^*n*^*Coef*_*i*_ × *X*_i_. “Coef” represents the regression coefficient of each gene set, “*X*” represents the ES of gene sets in the prognostic model, “*n*” represents the total number of gene sets in the prognostic model, and “*i*” represents the gene set that comprises the model. The patients were classified as high- and low-risk groups according to the median risk score. The relevant files generated during the calculation were uploaded as Supplementary Tables 6–11.

### 2.5. Statistical and Bioinformatic Analyses

Data analysis and graphical visualization were performed using R software (version 4.0.3, https://www.r-project.org) and GraphPad Prism (version 8.0). Heatmaps were used to show the ESs of gene sets of patients in various clusters and risk classifications. The hub genes were identified by constructing a protein-protein interaction (PPI) network in the STRING database.

The violin map is drawn by GraphPad Prism to show the gene expression differences of different groups. Kaplan-Meier survival curves and the log-rank test were used to perform survival analysis of patients in different groups (R package survival and survminer). The chi-square test and Fisher's exact test were used to analyze the differences in clinical features of patients in different groups. *P* < 0.05 was considered statistically significant, and all tests were two-tailed.

## 3. Results

### 3.1. Identification of Subtypes of AML Using Immunologic and Hallmark Gene Sets

**Figure 1:**
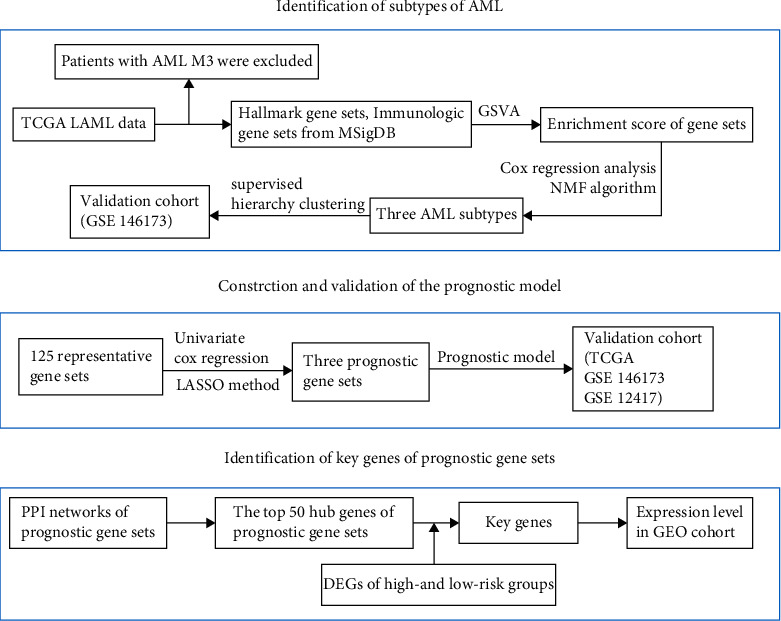
Research design and process diagram.

To investigate the difference in the activity of immunologic and hallmark gene sets in AML patients, the hallmark and C7 gene sets were downloaded from the MsigDB database. The study flowchart is illustrated in Figure 1. Hallmark gene sets are coherently expressed signatures that represent well-defined biological states or processes. C7 gene sets are immunologic signature gene sets, defined by microarray gene expression data from immunologic research.

**Figure 2:**
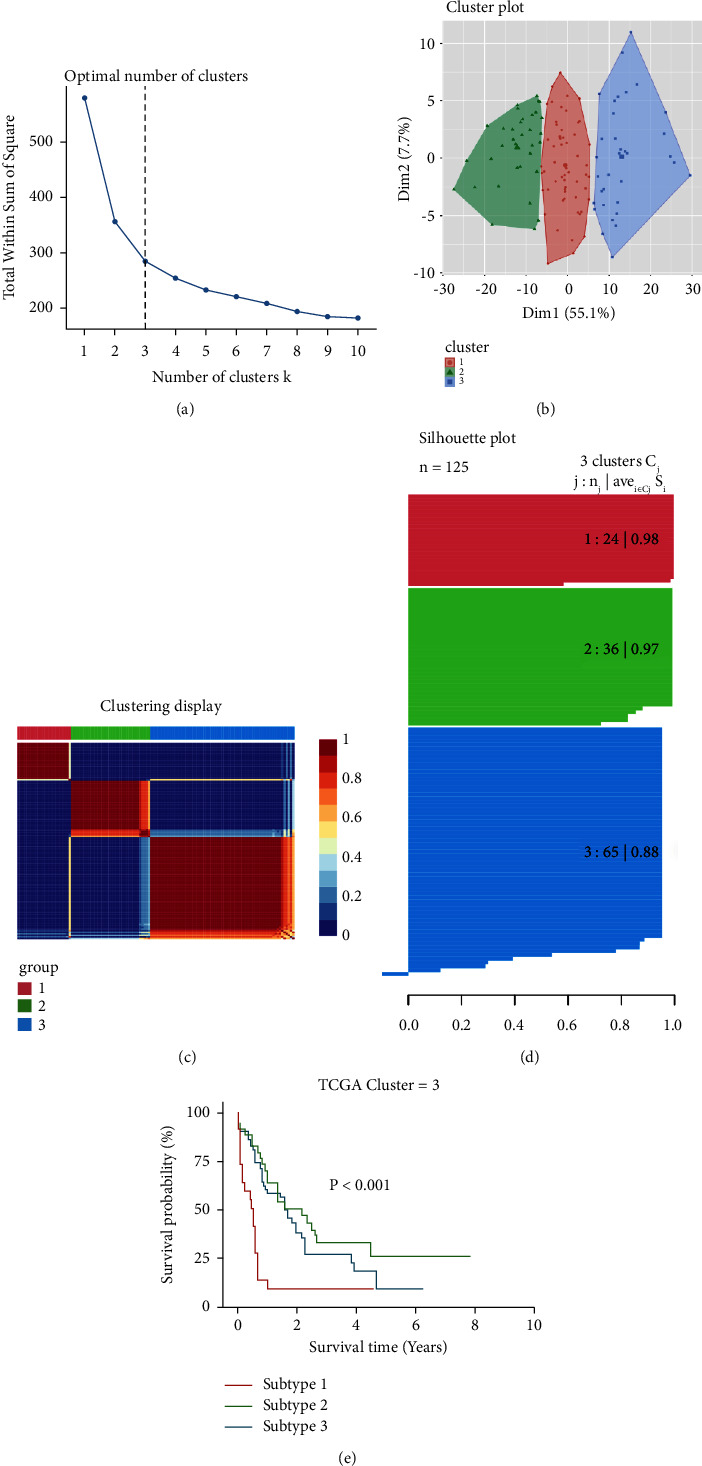
Identification of relevant subtypes of AML using immunologic and hallmark gene sets. (a) The optimal number of clusters (k) was determined with the factoextra package. (b) Visualization of cluster results, with k being 3. (c) The nonnegative matrix factorization (NMF) method was used to cluster AML samples. (d) The silhouette width plots displayed the clustering effect. The values of the silhouette range [−1, 1], where a high value indicates that the object is well matched to its own cluster and poorly matched to neighboring clusters. (e) Survival analysis among different subtypes.

**Figure 3:**
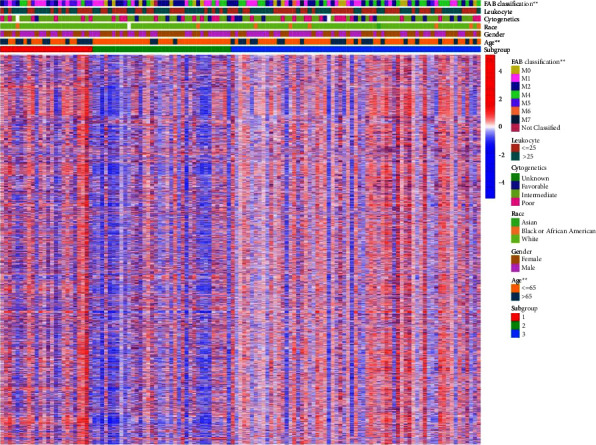
The heatmap displayed the correlation between the ES of gene sets and clinical characteristics (*P* < 0.001 “^*∗∗∗*^”; *P* < 0.01 “^*∗∗*^”; *P* < 0.05 “^*∗*^”).

Firstly, we obtained ES of immunologic and hallmark gene sets in the TCGA cohort (*n* = 125) with the GSVA method. Cox regression analysis was used for feature filtering through the R package CancerSubtype. The optimal number of clusters (K) was generated (*K* = 3, Figures 2(a) and 2(b)) using the factoextra package. AML patients are divided into three different subtypes (Figure 2(c)) by the NMF method, which is an effective dimension reduction method for cancer subtype identification. The silhouette width value was 0.92 in the silhouette width plots (Figure 2(d)), suggesting a fine match between an AML sample and its identified subtype. The patients with AML in subgroup 1 had worse overall survival than subgroups 2 and 3 (*P* < 0.001, Figure 2(e)), whose immunity was shown as a state of overall activation by heatmap (Figure 3). The median overall survival (mOS) of subgroups 1–3 was 0.4, 2.2, and 1.7 years, respectively. Moreover, age classification was significantly different among each subgroup (*P*=0.0012, Figure 3 and Table 1). In addition, the number of patients with M5 and M1 FAB classifications (37.5% and 29.17%, *P*=0.0044) was found to be more abundant in subtype 1 than in the other two subtypes.

### 3.2. Identification and Validation of Distinct Gene Sets of Three Subgroups

**Figure 4:**
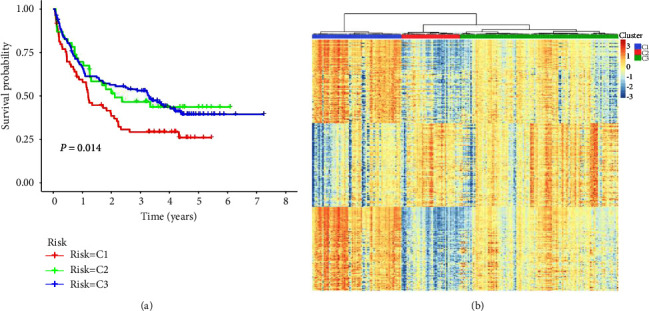
Identification of distinct gene sets of three subgroups in TCGA and their validation in GSE146173. (a) According to the distinct gene sets of the three subgroups in TCGA, the OS of patients in GSE146173 was distinguished by supervised hierarchical clustering. (b) The correlation between the ES of gene sets and clustering results of GSE146173 was shown.

According to the clustering results (Subtype1-3) of the training set (TCGA), the 100 most upregulated gene sets in each cluster (all gene sets were selected if they were less than 100) were used to conduct supervised hierarchical clustering in another gene expression profiling data (GSE146173) (the ES of immunologic and hallmark gene sets in the GEO cohort was displayed in Supplementary Table 4). As shown in Figure 4(a), patients with subtypes 2 and 3 had a more favorable prognosis than subtype 1 (*P* = 0.014). The heatmap displayed the ES of gene sets in each subtype in the GSE146173 cohort (Figure 4(b)). Similar to the TCGA cohort, the heatmap showed that most of the immune pathways of subtype 1 in the GSE146173 cohort tended to be “hot,” suggesting the activation state of overall immunity.

### 3.3. Construction and Validation of the Prognostic Model

**Figure 5:**
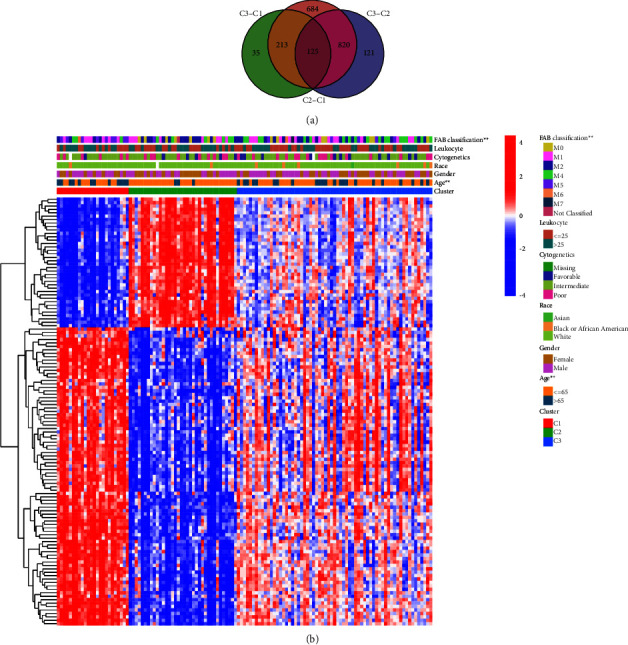
The representative gene sets in three subtypes. (a) The differential gene sets between each of the two subgroups were identified and intersected. (b) The heatmap showed the correlation between gene sets in the three subtypes and clinical characteristics.

**Figure 6:**
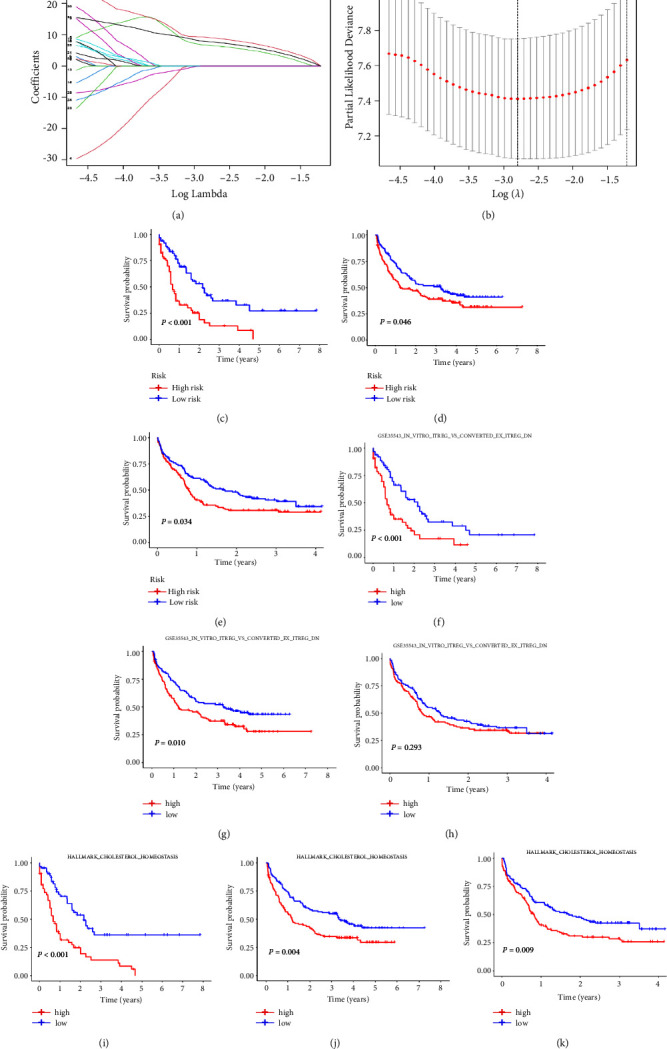
Prognostic model construction and validation. (a) LASSO coefficients of prognostic gene sets. (b) Three-fold cross-validation for the selection of prognostic gene sets in LASSO regression. Prognostic model was constructed based on LASSO regression analysis and validated in TCGA (c), GSE146173 (d), and GSE12417 cohorts (e). Patients in TCGA (f), GSE146173 (g), and GSE12417 (h) cohorts were divided into low- and high-risk groups according to the ES of GSE35543_IN_VITRO_ITREG_VS_CONVERTED_EX_ITREG_DN with median ES as the cutoff value. Patients in TCGA (i), GSE146173 (j), and GSE12417 (k) cohorts were divided into low- and high-risk groups according to the ES of HALLMARK_CHOLESTEROL_HOMEOSTASIS with median ES as the cutoff value.

To identify the representative gene sets that distinguish the three subgroups, we obtained the differential gene sets between each of the two subgroups and intersected them. One hundred and twenty-five representative gene sets were obtained (Figure 5(a) and Supplementary Table 6). As shown in Figure 5(b), ESs of 125 gene sets in the three subgroups were different. Univariate Cox regression was used to select the gene sets related to survival, and 62 gene sets remained for further analysis (Supplementary Table 7). Then we investigated prognostic gene sets with TCGA data by the LASSO regression method. The model with the minimum *λ* (0.06081) was selected as the best model, where the gene sets were reduced from 62 to 3 (Figures 6(a) and 6(b)). Thus, the gene sets of the final prognostic model and their corresponding coefficients were obtained (Supplementary Table 8). The risk score is defined as follows: risk score = ES of GSE36891_UNSTIM_VS_PAM_TLR2_STIM_PERITONEAL_MACROPHAGE_DN × 6.63193270349141 + ES of GSE35543_IN_VITRO_ITREG_VS_CONVERTED_EX_ITREG_DN × 9.1162940208133 + ES of HALLMARK_CHOLESTEROL_HOMEOSTASIS × 7.78597962602926.

The patients were divided into high- and low-risk groups, with the median score as the cutoff value. Patients in the high-risk group had a poorer prognosis than those in the low-risk group in the TCGA cohort (*P* < 0.001, Figure 6(c)). The mOS of low- and high-group was 2.2 and 0.7 years, respectively. Subsequently, the prognostic model was further validated in GSE146173 (*P* = 0.046, Figure 6(d)) and GSE12417 (*P* = 0.034, Figure 6(e)). The mOS of the low- and high-group was 3.2 and 1.2 years, respectively, in the GSE146173 cohort; in the GSE12417 cohort, the mOS of the low- and high-group was 1.7 and 0.8 years, respectively. The clinical characteristics of two risk groups in three cohorts were analyzed (Supplementary Tables 12–14). We further investigated the prognostic value of each gene set. The results showed that a high ES of HALLMARK_CHOLESTEROL_HOMEOSTASIS (Figures 6(i)–6(k)) was correlated with short overall survival in the TCGA, GSE146173, and GSE12417 cohorts. Patients with high ES of GSE35543_ IN_ VITRO_ ITREG_ VS_ CONVERTED_ EX_ ITREG_ DN (Figure 6(f)) had a poor prognosis in the TCGA and GSE146173 cohort but not in the GSE12417 cohort. The high ES of GSE36891_UNSTIM_VS_PAM_TLR2_STIM_PERITONEAL_MACROPHAGE_DN was only associated with poor prognosis in the TCGA cohort (Supplementary Figures 1(a)–1(c)).

### 3.4. Exploration of Key Genes of Prognostic Gene Sets

**Figure 7:**
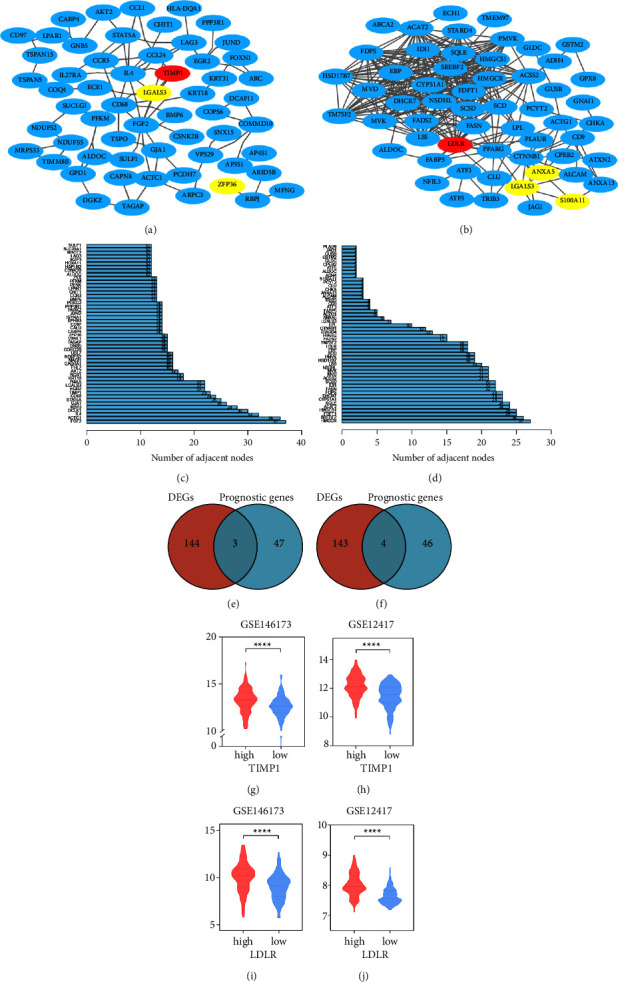
Identification of key genes in prognostic gene sets. The PPI network of GSE35543_IN_VITRO_ITREG_VS_CONVERTED_EX_ITREG_DN (a) and HALLMARK_CHOLESTEROL_HOMEOSTASIS (b). The top 50 genes with the most nodes in the gene sets GSE35543_IN_VITRO_ITREG_VS_CONVERTED_EX_ITREG_DN and HALLMARK_CHOLESTEROL_HOMEOSTASIS are shown in (c) and (d), respectively. The DEGs of the high- and low-risk groups were obtained and intersected with the 50 candidate genes in each gene set (e, f). (g–j) The gene expression levels of TIMP1 and LDLR in GSE146173 and GSE12417.

The PPI networks of GSE35543_IN_VITRO_ITREG_VS_CONVERTED_EX_ITREG_DN (Figure 7(a)) and HALLMARK_CHOLESTEROL_HOMEOSTASIS (Figure 7(b)) were built through the String website, and the results were imported into Cytoscape for further analysis. The top 50 genes were selected based on the ranking of gene-connecting nodes (Figures 7(c) and 7(d) and Supplementary Table 15). To further identify the key genes that have an influence on the prognosis, we intersected the differentially expressed genes (DEGs) between the high- and low-risk groups in the TCGA cohort (*P* < 0.05 and |log FC| > 1, Supplementary Table 16) with the 50 candidate genes from gene sets GSE35543_IN_VITRO_ITREG_VS_CONVERTED_EX_ITREG_DN (Figure 7(e)) and HALLMARK_CHOLESTEROL_HOMEOSTASIS (Figure 7(f)), respectively. For the gene set GSE35543_IN_VITRO_ITREG_VS_CONVERTED_EX_ITREG_DN, three intersection genes were obtained, including TIMP1, ZFP36, and LGALS3. For gene set HALLMARK_CHOLESTEROL_HOMEOSTASIS, four intersection genes were obtained, which were LDLR, LGALS3, S100A11, and ANXA5. Amongst these genes, TIMP1 and LDLR have the largest number of nodes in the two gene sets. As shown in Figures 7(g)–7(j), TIMP1 and LDLR were significantly increased in the high-risk groups in the two GEO cohorts (*P* < 0.0001), which further confirms the ability of the prognostic model to distinguish patients between different risks and provides clues for further research.

## 4. Discussion

AML is a malignant myeloid neoplasm with a significant amount of heterogeneity in tumor biology and poor clinical outcomes. Traditional analysis strategies have focused on comparing the differential gene expression between two groups of interested populations. However, some pathways may be significantly regulated without a significant change in single gene expression. To meet this challenge, we applied GSVA to display the activity changes of each pathway in immunologic and hallmark gene sets in AML samples. According to the ESs of immunologic and hallmark gene sets in TCGA samples, AML patients were clustered into three subtypes with the NMF algorithm. Patients with subtypes 2 and 3 had a more favorable prognosis than patients with subtype 1, which was validated in the GSE146173 cohort. Subsequently, one hundred and twenty-five differential gene sets were further screened using univariate Cox regression and LASSO regression methods, and 3 prognostic gene sets were identified. Based on these three gene sets, we constructed the final prognosis model and divided the patients into high-risk and low-risk groups in TCGA, GSE146173, and GSE12417 cohorts.

Our results identified three prognostic gene sets, and they were related to macrophages, Treg, and cholesterol homeostasis, respectively. In the past decade, increasing evidence revealed the role of macrophages in the etiology and progression of both solid tumors and blood malignancies. Macrophages in solid tumor tissue and the bone marrow microenvironment are referred to as tumor-associated macrophages (TAMs) and leukemia-associated macrophages (LAMs), respectively. Based on their functional phenotypes, macrophages are divided into two phenotypes, M1 and M2. In brief, M1 macrophages have antitumor effects, whereas M2 is believed to promote tumor progression [11]. Yang et al. revealed that the acquisition of M1 characteristics in leukemia-associated macrophages (LAMs) through activation of the IRF7-SAPK/JNK pathway was associated with prolonged survival in mice. Besides, the high-level expression of CD163, which is the typical marker of M2 macrophages, was associated with worse overall survival in AML patients [12].

Previous studies have shown that the cholesterol synthesis of AML cells increased significantly after being exposed to chemotherapy [13]. Inhibiting cholesterol synthesis can kill AML cells and sensitize them to chemotherapeutic drugs [14, 15], suggesting that AML cells require higher levels of cholesterol to maintain survival compared with normal cells and that the imbalance of cholesterol homeostasis may lead to treatment failure. A phase 2 study of pravastatin in combination with idarubicin and cytarabine reported an encouraging response rate for relapsed AML [16] and improved overall survival for patients in the low-risk group [17]. Cholesterol homeostasis plays an important role in the survival of AML cells and eventually affects the prognosis of patients with AML.

Regulatory T cells (Tregs) are considered to play a key role in immune suppression and angiogenesis via secreting immunosuppressive cytokines and molecules, performing cytolytic functions, disrupting metabolism, and attenuating the capacity of dendritic cells (DCs) [18]. Patients with AML had a higher frequency of Tregs in peripheral blood and bone marrow than healthy participants [19, 20], while patients who achieved complete remission had a comparable frequency of Tregs compared with healthy controls. Wan et al. revealed that Tregs lead to immune escape by inhibiting the function of CD4+CD25− T cells [19]. These results indicate that Tregs are involved in the tumorigenesis of AML.

Amongst three prognostic gene sets, high ES of HALLMARK_CHOLESTEROL_HOMEOSTASIS and GSE35543_ IN_ VITRO_ ITREG_ VS_ CONVERTED_ EX_ ITREG_ DN was correlated with short overall survival in at least two cohorts. We further investigated the key genes in these gene sets. The key genes of GSE35543_ IN_ VITRO_ ITREG_ VS_ CONVERTED_ EX_ ITREG_ DN included TIMP1, ZFP36, and LGALS3. The key genes of HALLMARK_CHOLESTEROL_HOMEOSTASIS included LDLR, LGALS3, S100A11, and ANXA5. Amongst these genes, TIMP1 and LDLR have the largest number of nodes in the PPI network of these two gene sets. TIMP1 and LDLR have been reported to be associated with the prognosis of AML [21–23].

There were several limitations in this study. First, our data were acquired from the TCGA database, which lacked important clinical information, including treatment regimens and event-free survival, limiting further investigation of the prognostic model. Second, the prognostic model was conducted using publicly accessible datasets and requires multicenter clinical samples to assess its prognostic value.

## 5. Conclusion

Briefly, the study provided a full view of activity changes in hallmark and immunologic gene sets and established a novel prognostic model for risk stratification in AML, which provides a new perspective for optimizing prognostic assessment strategies in the future.

## Figures and Tables

**Table 1:** Analysis of clinical characteristics in three subtypes.

	Subgroup 1	Subgroup 2	Subgroup 3	*P*
Age (years), *n* (%)				
≤65	12 (50)	33 (91.67)	42 (64.62)	0.0012
>65	12 (50)	3 (8.33)	23 (35.38)	
Gender, *n* (%)				
Female	9 (37.5)	17 (47.22)	29 (44.62)	0.7508
Male	15 (62.5)	19 (52.78)	36 (55.38)	
Race, *n* (%)				
Asian	0 (0.0)	0 (0.0)	1 (1.54)	0.5978
Black or African American	1 (4.17)	1 (2.86)	6 (9.23)	
White	23 (95.83)	34 (97.14)	58 (89.23)	
Cytogenetics, *n* (%)				
Favorable	1 (4.17)	5 (13.89)	11 (17.19)	
Intermediate	16 (66.67)	22 (61.11)	37 (57.81)	
Poor	6 (25)	9 (25)	16 (25)	
Unknown	1 (4.17)	0 (0.0)	0 (0.0)	0.3639
Leukocyte (×10^9/L), *n* (%)				
≤25	11 (45.83)	18 (50)	37 (56.92)	0.5993
>25	13 (54.17)	18 (50)	28 (43.08)	
FAB subtype, *n* (%)				
M0	0 (0)	7 (19.44)	7 (10.77)	0.0044
M1	7 (29.17)	8 (22.22)	15 (23.08)	
M2	4 (16.67)	13 (36.11)	17 (26.15)	
M4	3 (12.5)	6 (16.67)	19 (29.23)	
M5	9 (37.5)	1 (2.78)	5 (7.69)	
M6	1 (4.17)	0 (0)	1 (1.54)	
M7	0 (0)	0 (0)	1 (1.54)	
Not classified	0 (0)	1 (2.78)	0 (0)	

FAB, French-American-British.

## Data Availability

The datasets analyzed in this study are available in the GEO database (http://www.ncbi.nlm.nih.gov/geo/) and TCGA (https://portal.gdc.cancer.gov/repository). The datasets generated during the current study are available from the corresponding author on reasonable request.

## Abbreviations

AML: Acute myeloid leukemia
GSVA: Gene set variation analysis
NMF: Nonnegative matrix factorization
ES:Enrichment scoreLASSO: Enrichment score LASSO: Least absolute shrinkage and selection operator
OS: Overall survival
ELN: European leukemia net
FPKM: Fragments per kilobase per million
PPI: Protein-protein interaction
FAB: French-American-British subclassification.

## Supplementary Materials

Supplementary Materials
*Supplementary Table 1.*The enrichment score (ES) of immunologic and hallmark gene sets in the TCGA cohort by gene set variation analysis (GSVA). *Supplementary Table 2.* The subtypes of AML patients in the TCGA cohort with the nonnegative matrix factorization (NMF) method. *Supplementary Table 3.* The differences in the enrichment score (ES) of immunologic and hallmark gene sets among each cluster. *Supplementary Table 4.* The enrichment score (ES) of immunologic and hallmark gene sets in the GSE146173 cohort. *Supplementary Table 5.* The subtypes of AML patients in the GSE146173 cohort by supervised hierarchical clustering. *Supplementary Table 6.* One hundred and twenty-five representative gene sets whose ES differed among the three subgroups. *Supplementary Table 7.*Sixty-two gene sets that were related to survival by univariate Cox regression analysis. *Supplementary Table 8.* The gene sets constructing the final prognostic model and their corresponding coefficients. *Supplementary Table 9.* The risk category of patients in the TCGA cohort based on the prognostic model. *Supplementary Table 10.* The risk category of patients in the GSE146173 cohort based on the prognostic model. *Supplementary Table 11.* The risk category of patients in the GSE12417 cohort based on the prognostic model*. Supplementary Table 12*. Analysis of clinical characteristics in the TCGA cohort. *Supplementary Table 13.* Analysis of clinical characteristics in the GSE146173 cohort. *Supplementary Table 14*. Analysis of clinical characteristics in the GSE12417 cohort*. Supplementary Table 15*. The top 50 genes based on the ranking of gene-connecting nodes. *Supplementary Table 16.* The differentially expressed genes (DEGs) between the high- and low-risk groups in the TCGA cohort. *Supplementary Figure 1*. Patients in TCGA (A), GSE146173 (B), and GSE12417 (C) cohorts were divided into low- and high-risk groups according to the ES of GSE36891_UNSTIM_VS_PAM_TLR2_STIM_PERITONEAL_MACROPHAGE_DN, with the median ES as the cutoff value.
