# Supplementary figures and images for: A Novel Prognostic Model for Acute Myeloid Leukemia Based on Gene Set Variation Analysis
Source: J Oncol. 2022 Nov 21;2022:7727424. doi: 10.1155/2022/7727424 (PMC9705098; doi:10.1155/2022/7727424)

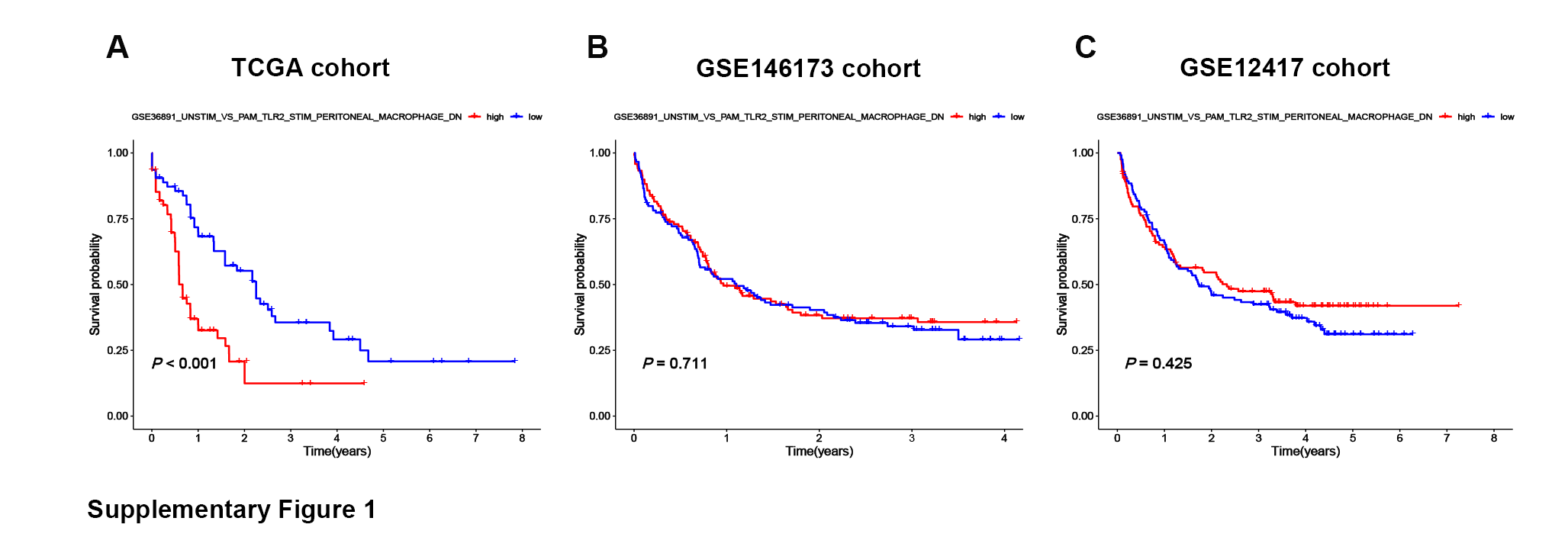

Supplement: Supplementary Materials — Supplementary Table 1.The enrichment score (ES) of immunologic and hallmark gene sets in the TCGA cohort by gene set variation analysis (GSVA). Supplementary Table 2. The subtypes of AML patients in the TCGA cohort with the nonnegative matrix factorization (NMF) method. Supplementary Table 3. The differences in the enrichment score (ES) of immunologic and hallmark gene sets among each cluster. Supplementary Table 4. The enrichment score (ES) of immunologic and hallmark gene sets in the GSE146173 cohort. Supplementary Table 5. The subtypes of AML patients in the GSE146173 cohort by supervised hierarchical clustering. Supplementary Table 6. One hundred and twenty-five representative gene sets whose ES differed among the three subgroups. Supplementary Table 7.Sixty-two gene sets that were related to survival by univariate Cox regression analysis. Supplementary Table 8. The gene sets constructing the final prognostic model and their corresponding coefficients. Supplementary Table 9. The risk category of patients in the TCGA cohort based on the prognostic model. Supplementary Table 10. The risk category of patients in the GSE146173 cohort based on the prognostic model. Supplementary Table 11. The risk category of patients in the GSE12417 cohort based on the prognostic model. Supplementary Table 12. Analysis of clinical characteristics in the TCGA cohort. Supplementary Table 13. Analysis of clinical characteristics in the GSE146173 cohort. Supplementary Table 14. Analysis of clinical characteristics in the GSE12417 cohort. Supplementary Table 15. The top 50 genes based on the ranking of gene-connecting nodes. Supplementary Table 16. The differentially expressed genes (DEGs) between the high- and low-risk groups in the TCGA cohort. Supplementary Figure 1. Patients in TCGA (A), GSE146173 (B), and GSE12417 (C) cohorts were divided into low- and high-risk groups according to the ES of GSE36891_UNSTIM_VS_PAM_TLR2_STIM_PERITONEAL_MACROPHAGE_DN, with the median ES as the cutoff [file 7727424.f1.zip › Supplementary Figure 1.tif]
